# Empagliflozin in heart failure patients with reduced ejection fraction: a randomized clinical trial (Empire HF)

**DOI:** 10.1186/s13063-019-3474-5

**Published:** 2019-06-21

**Authors:** Jesper Jensen, Massar Omar, Caroline Kistorp, Mikael Kjær Poulsen, Christian Tuxen, Ida Gustafsson, Lars Køber, Finn Gustafsson, Emil Fosbøl, Niels Eske Bruun, Lars Videbæk, Peter Hartmund Frederiksen, Jacob Eifer Møller, Morten Schou

**Affiliations:** 1 Department of Cardiology, Herlev-Gentofte Hospital, Herlev Ringvej 75, 2730 Herlev, DK Denmark; 2 Department of Cardiology, Odense University Hospital, J. B. Winsløws Vej 4, 5000 Odense C, DK Denmark; 3 Department of Endocrinology, Rigshospitalet, Blegdamsvej 9, 2100 København Ø, DK Denmark; 4 Department of Cardiology, Bispebjerg-Frederiksberg Hospital, Nordre Fasanvej 57, 2000 Frederiksberg, DK Denmark; 5 Department of Cardiology, The Heart Centre, Rigshospitalet, Blegdamsvej 9, 2100 København Ø, DK Denmark; 6 Department of Cardiology, Zealand University Hospital, Sygehusvej 10, 4000 Roskilde, DK Denmark; 7 Faculty of Health and Medical Sciences, Copenhagen University, Blegdamsvej 3B, 2200 København N, DK Denmark; 8 Faculty of Health Sciences, University of Southern Denmark, J.B. Winsløws Vej 19, 3, 5000 Odense C, DK Denmark; 9 Clinical Institute, Aalborg University, Søndre Skovvej 15, 9000 Aalborg, DK Denmark

**Keywords:** Heart failure, SGLT2 inhibitors, Mechanism, Mode of action, NT-proBNP, Daily activity level, Cardiac function, Metabolic endpoints, Renal endpoints, Quality of life

## Abstract

**Background:**

Data from recent cardiovascular outcome trials in patients with type 2 diabetes (T2D) suggest that sodium-glucose cotransporter 2 (SGLT2) inhibitors can prevent development of heart failure (HF) and prolong life in patients without HF. Ongoing event-driven trials are investigating whether the same effect is present in patients with well-defined HF. The mechanism behind the effect of SGLT2 inhibitors in patients with T2D and the potential effect in patients with overt HF is presently unknown.

**Methods:**

This is a randomized, double-blinded, placebo-controlled, parallel group, clinical trial including HF patients with reduced left ventricular ejection fraction (HFrEF) with an ejection fraction ≤ 40% on optimal therapy recruited from specialized HF clinics in Denmark. The primary aim is to investigate the effect of the SGLT2 inhibitor empagliflozin on N-terminal pro-brain natriuretic peptide (NT-proBNP). Secondary endpoints include cardiac biomarkers, function and hemodynamics, metabolic and renal parameters, daily activity level, and quality of life. Patients are assigned 1:1 to 90 days treatment with empagliflozin 10 mg daily or placebo. Patients with T2D are required to be on recommended doses of anti-glycemic therapy with a hemoglobin A1c (HbA1c) of 6.5–10.0% (48–86 mmol/mol). To show a between-group difference in the change of NT-proBNP of 30%, a total of 189 patients will be included.

**Discussion:**

The Empire HF trial will elucidate the effects and modes of action of empagliflozin in HFrEF patients with and without T2D and provide important mechanistic data which will complement ongoing event-driven trials.

**Trial registration:**

Clinicaltrialsregister.eu, EudraCT Number 2017-001341-27. Registered on 29 May 2017.

ClinicalTrials.gov, NCT03198585. Registered on 26 June 2017.

**Electronic supplementary material:**

The online version of this article (10.1186/s13063-019-3474-5) contains supplementary material, which is available to authorized users.

## Background

Within recent years, attention to heart failure (HF) care in patients with type 2 diabetes (T2D) has increased markedly after results from three randomized clinical trials (RCT) evaluating the effect of sodium-glucose co-transporter 2 (SGLT2) inhibitors [1–3]. In these safety trials, it was observed that three different SGLT2 inhibitors could prevent development of HF and prolong life in patients with T2D. Recently, the results have been replicated in real life [4, 5]. The mechanism behind these observations are poorly understood and while several hypotheses have been proposed, data are lacking [6–9].

In the Empagliflozin, Cardiovascular Outcomes, and Mortality in Type 2 Diabetes (EMPA-REG OUTCOME) trial, the Canagliflozin Cardiovascular Assessment Study (CANVAS) program, and the Dapagliflozin Effect on Cardiovascular Events-Thrombolysis in Myocardial Infarction 58 (DECLARE-TIMI 58) trial, 10–14% of the included patients had a reported history of HF at baseline and a significant subgroup effect was observed with a reduction in the composite endpoint of hospitalization for HF or cardiovascular death in patients treated with empagliflozin, canagliflozin, and dapagliflozin, respectively [3, 10, 11]. However, HF was neither well-defined nor described in these trials; central information such as whether the patients with reported HF had preserved (HFpEF) or reduced (HFrEF) ejection fraction is unknown. The possible beneficial effects of SGLT2 inhibitors are unknown in patients without T2D but the primary action in the kidneys – induction of glucosuria and natriuresis – have been demonstrated in individuals without T2D [12]. Proposed derived mechanisms potentially explaining the observed cardiovascular benefits of SGLT2 inhibitors include a proportionally larger reduction in the extracellular volume (ECV) than in the plasma volume (PV) [13, 14], leading to a reduced preload, left ventricular (LV) filling pressure, and LV wall stress, the latter being the primary driver for N-terminal pro-brain natriuretic peptide (NT-proBNP) production. Thereby, a net effect of decreased NT-proBNP levels, which is associated with a decreased mortality risk in HF populations, is plausible [15, 16] and a decrease of 30% in NT-proBNP has previously been shown to be clinically significant in this population [17]. Other proposed derived mechanisms of SGLT2 inhibitors supporting beneficial cardiovascular effects in HF patients include a favorable shift in glucose and fat metabolism towards increased ketone substrate use [18, 19], renoprotective effects with changes in intrarenal hemodynamics, uricosuria and reduced albuminuria [7], and direct cardiac effects with remodeling of the myocardium ultimately leading to an improvement in the ventricular systolic function [9].

To increase the understanding of the cardiovascular effects of SGLT2 inhibitors, we are evaluating the effect of the SGLT2 inhibitor empagliflozin in HFrEF patients with a left ventricular ejection fraction (LVEF) ≤ 40% on cardiac biomarkers, function and hemodynamics, metabolic and renal parameters, and daily activity level and quality of life. The results of the Empire HF trial will complement the results from ongoing event-driven trials and may provide pathophysiological insight into the effect of this new group of drugs in HFrEF patients. Here, we present the protocol for the Empire HF trial. A checklist in accordance with the Standard Protocol Items: Recommendations for Interventional Trials (SPIRIT) for the reporting of the protocol is available (Additional file 1).

## Methods

### Study hypotheses

**Fig. 1:**
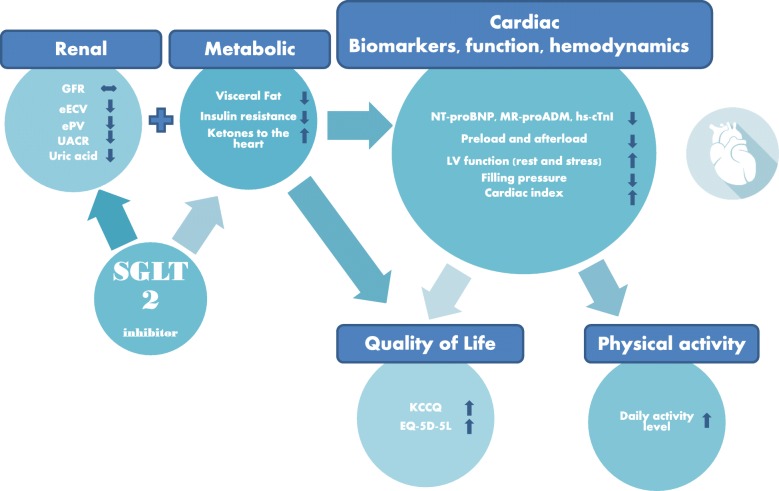
Empire HF hypotheses. SGLT2 sodium-glucose cotransporter 2, GFR glomerular filtration rate, eECV estimated extracellular volume, ePV estimated plasma volume, UACR urine albumin to creatinine ratio, NT-proBNP N-terminal pro-brain natriuretic peptide, MR-proADM mid-region pro-peptide of adrenomedullin, hs-cTNI high-sensitivity cardiac troponin I, LV left ventricular, KCCQ Kansas City cardiomyopathy questionnaire, EQ-5D-5 L EuroQol 5-dimension 5-level

The study hypotheses are presented in Fig. 1. The main hypothesis of the Empire HF trial is that 90 days treatment with empagliflozin 10 mg once daily compared with placebo reduces NT-proBNP in stable HFrEF patients on optimal therapy. An exploratory hypothesis is that the treatment is associated with an increase in the daily activity level and secondary hypotheses include that the treatment: reduces the amount of visceral fat, reduces insulin resistance, and increases supply of ketones to the heart (*Metabolic hypothesis*); maintains glomerular filtration rate (GFR) and reduces estimated extracellular volume (eECV), estimated plasma volume (ePV), uric acid, and urinary excretion of albumin (*Renal hypothesis*); reduces plasma concentrations of mid-region pro-peptide of adrenomedullin (MR-proADM) and high-sensitivity cardiac troponin I (hs-cTnI) (*Cardiac Biomarker hypothesis*); improves left ventricular global longitudinal strain (LV-GLS) and LVEF at rest and during pharmacological stress (*Cardiac function hypothesis*); reduces the pulmonary capillary wedge pressure (PCWP) to cardiac index (CI) ratio during sub-maximal exercise and improves LV contractile reserve (*Hemodynamic hypothesis*); improves health-related quality of life (*Quality of life hypothesis*).

### Primary objective

The primary objective of the study is to assess the effect of empagliflozin on NT-proBNP.

### Secondary objectives

An exploratory objective of the study is to assess the effect of empagliflozin on daily activity level. Secondary objectives include assessment of the effects on body composition, glucose metabolism, and ketones; GFR, eECV, ePV, urid acid, and urine albumin to creatinine ratio (UACR); cardiac biomarkers including MR-proADM and hs-cTnI; LV diastolic and systolic function at rest and during low-dose dobutamine infusion with echocardiography; central invasive hemodynamics at rest and during exercise; and health-related quality of life.

### Study design

Investigator-initiated, double-blinded, placebo-controlled, parallel group RCT. Two experimental sites and five recruiting sites.

### Study population

Stable outpatients with HFrEF on optimal therapy in accordance with most recent European and national guidelines [20]. It is expected that 20% of the patients have known T2D and that an additional 30% of the patients will have a diagnosis of new onset T2D or impaired glucose tolerance based on an oral glucose tolerance test (OGTT) at the randomization visit [21, 22].

### Primary endpoint

Between-group difference in the change of plasma concentrations of NT-proBNP from baseline to 90 days.

### Secondary endpoints

All the below secondary endpoints will be quantified as the between-group difference in the change of the given endpoint from baseline to 90 days. As an exploratory endpoint, the amount of daily average accelerometer units is chosen, measured by patient-worn accelerometers [23]. Secondary endpoints include: visceral fat assessed by whole-body dual-energy X-ray absorptiometry (DXA) scan [24]; ePV assessed by hematocrit and hemoglobin [25]; glucose metabolism assessed by OGTT including assessment of insulin sensitivity quantified with the Matsuda index [21]; ketone supply to the heart assessed by measuring beta-hydroxybutyrate [26, 27]; renal function assessed by measuring GFR and eECV with chromium-51 labelled ethylenediamine tetraacetic acid (^51^Cr-EDTA) clearance [28, 29]; uric acid and UACR [30, 31]; cardiac biomarkers assessed by measuring MR-proADM and hs-cTnI [32, 33]; cardiac systolic and diastolic function including LV-GLS and LVEF assessed by transthoracic echocardiography at rest and during low-dose dobutamine stress with a dosage of 20 μg/kg/min [34, 35]; cardiac hemodynamics during sub-maximal exercise assessed by right heart catheterization (RHC) including PCWP/CI index and LV contractile reserve [36]; and health-related quality of life assessed by the questionnaires Kansas City Cardiomyopathy Questionnaire (KCCQ) and EuroQol 5-dimension 5-level (EQ-5D-5 L) questionnaire [37, 38].

### Assessment and randomization

**Fig. 2:**
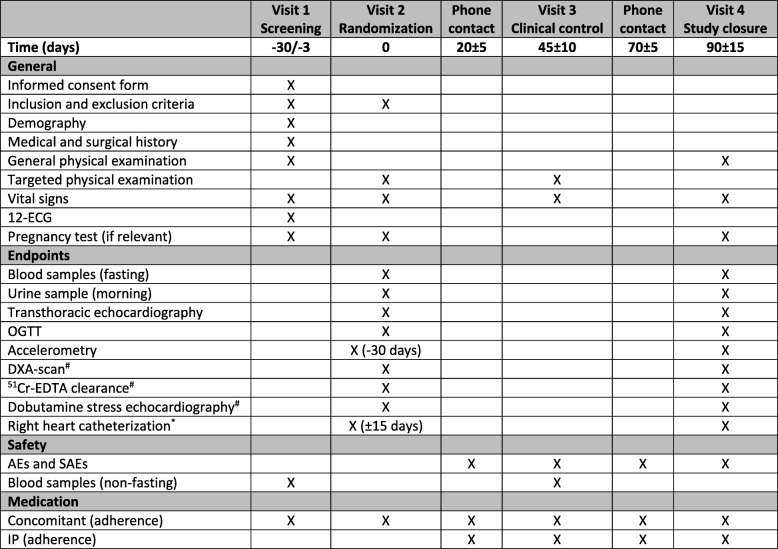
Schedule of enrolment, interventions, and assessments in accordance with the Standard Protocol Items: Recommendations for Interventional Trials (SPIRIT). ^#^Only performed in the patients enrolled at Herlev-Gentofte Hospital. ^*^Only performed in a sub-group of the patients enrolled at Odense University Hospital. ECG electrocardiogram, OGTT oral glucose tolerance test, DXA dual-energy X-ray absorptiometry, ^51^Cr-EDTA chromium-51 labeled ethylenediamine tetra-acetic acid. AE adverse event, SAE severe adverse event, IP investigational product

The schedule of enrolment, interventions, and assessments in accordance with SPIRIT is outlined in Fig. 2. At the screening visit, informed consent is obtained by an investigator or trained study nurse, baseline characteristics assessed based on the medical record and confirmed in consultation with the patient, and eligibility confirmed with fulfilment of all inclusion criteria and no exclusion criteria. At the following randomization visit, patients are randomized 1:1 regarding both the primary and secondary endpoints. To ensure both safety assessment, adherence, and complete follow-up, follow-up assessments are carried out as two telephone contacts, a control visit, and an end-of-study visit. The allocated treatment of individual patients may be discontinued or modified based on the patient’s decision, a positive pregnancy test, severe non-adherence, or if the investigators assess that further treatment is contraindicated because of adverse events (AEs) or other safety reasons. If possible, patients who discontinue or deviate from the protocol will be followed up regarding all endpoints. Strategies to improve adherence include information at both the screening and randomization visit regarding correct drug administration and the patients are encouraged to use a medication diary or pill boxes during the study. At all planned contacts during the treatment period, the patients are asked whether they adhere to the correct drug administration. At the end of the study visit, excess tablets of the investigational product (IP) are returned for drug accountability.

### Inclusion and exclusion criteria

Inclusion and exclusion criteria are presented in Table 1. Both HF patients with and without T2D are included. Patients with known T2D will be treated in accordance with European and national guidelines and will be on recommended, stable dose(s) of anti-glycemic drug(s) for 30 days before randomization and no additional anti-glycemic drugs will be added during the study period. Patients with new onset T2D at the randomization visit will not receive additional anti-glycemic treatment during the study period. Regarding treatment for HF, patients will be on optimal, stable medical treatment in accordance with European guidelines for 30 days before randomization. If indicated, a device will be implanted before randomization in accordance with national guidelines. For implantation of a cardioverter defibrillator (ICD) and cardiac resynchronization therapy (CRT), a minimum period of 30 and 90 days will be obtained between implantation and randomization, respectively. During the study period, conventional HF therapy will only be changed if side effects are suspected.

**Table 1:** Inclusion and exclusion criteria

Inclusion criteria	Exclusion criteria
Optimal heart failure therapy in accordance with European and national guidelines	CRT-D/−P implanted < 90 days
LVEF ≤ 0.40	Uncorrected severe valvular disease
eGFR > 30 mL/min/1.73 m^2^	Non-compliance
BMI < 45 kg/m^2^	Use of metalozone
NYHA class I–III	NYHA class IV
Age > 18 years	Age > 85 years
	Dementia
	Admission for HF < 30 days
If T2D – optimal treatment in accordance with European and national guidelines	Admission for hypoglycemia < 12 months
If T2D – stable doses of anti-glycemic treatment for 30 days	Known sustained VT
If T2D – HbA1c 6.5–10.0%	Symptomatic hypotension and systolic BP < 95 mmHg
	Unable to perform an exercise test
	Immobilization
	Pregnancy
	Participation in other medical trials
	Previous intolerance of Empagliflozin or excipients

*LVEF* left ventricular ejection fraction, *eGFR* estimated glomerular filtration rate, *BMI* body mass index, *NYHA* New York Heart Association, *T2D* type 2 diabetes, *HbA1c* hemoglobin A1c, *CRT-D/−P* cardiac resynchronization therapy with defibrillator (−D) or without defibrillator (−P), *HF* heart failure, *VT* ventricular tachycardia, *BP* blood pressure

### Schedule of enrolment, interventions, and assessments

Eligible patients will undergo the study visits presented in Fig. 2. In addition, unlimited access to telephone and email service is available and, if considered necessary, unscheduled visits during the study period through four weeks after the end-of-study visit is planned. Follow-up will be performed by investigators, sub-investigators, and study nurses educated in the specialized treatment of HF patients and who are trained in the study protocol. Important protocol modifications will be communicated to relevant parties including regulators, the ethical review board, investigators, trial participants, trial registries, and journals.

### Statistical analyses

Intention-to-treat (ITT) analyses will be applied as the primary analysis. Analysis of the primary endpoint is comparison of the between-group difference in the change of NT-proBNP from baseline to 90 days. The primary endpoint will be analyzed using analysis of covariance (ANCOVA), with treatment as a fixed factor, the baseline NT-proBNP level as a covariate and with adjustment for age, sex, history of T2D, and site of randomization. Missing data in the primary analysis will be estimated using imputation. Approximately half the patients will have either known T2D, new-onset T2D, or impaired glucose tolerance. Test for interaction between treatment, abnormal glucose tolerance, and all the specified endpoints (predefined sub-group analyses) will be performed. Log transformation will be performed if assumption of normality is not met. After log transformation, the parameter will be further tested for normality as indicated. A two-tailed *p* value ≤ 0.05 is considered statistically significant. Normally distributed variables will be presented as mean ± standard deviation (SD) and skewed distributed variables as median and interquartile range [IQR]. Comparisons between treatment and placebo group will be performed by an unpaired two sample *t-*test, Mann–Whitney test, or χ^2^ test as appropriate.

### Sample size

Primary endpoint: Based on data from a previous study, it is expected that a reduction in NT-proBNP of 30% is clinically significant [17]. To test the primary hypothesis that empagliflozin 10 mg daily compared with placebo reduces NT-proBNP with 30% (SD of 70%) with a power of 0.80 and a significance level of 0.05, a total of *N* = 172 patients in the main study is required. To allow for dropouts, the final total sample size is planned to be 189 patients.

Low dose dobutamine sub-study: To detect a clinically significant decrease in LV-GLS of 20% (SD of 30%) in patients treated with empagliflozin compared with placebo in this secondary endpoint with a power of 0.80 and a significance level of 0.05, a total of 72 patients are required. To allow for dropouts, *N* = 119 patients are planned to be enrolled at Herlev-Gentofte Hospital in this sub-study (*Cardiac function hypothesis*).

Hemodynamic sub-study: PCWP is expected to increase to 32 ± 8 mmHg and CI to 5.0 ± 1.4 L/min/m^2^ with exercise, with a PCWP/CI ratio equal to 6.4 ± 1.4. To detect a decrease of 20% in patients treated with empagliflozin compared with placebo in this secondary endpoint with a power of 0.80 and a significance level of 0.05, a total of 61 patients are required. To allow for dropouts, *N* = 70 patients are planned to be enrolled at Odense University Hospital in this sub-study (*Hemodynamic hypothesis*).

In all the other sub-studies, the sample size is a consequence of the main study on NT-proBNP and confidence intervals will be evaluated critically. Based on previous studies it is expected to observe a possible significant difference with the used sample sizes [24, 39].

### Blinding

Both patients and investigators are blinded to the treatment allocation. The IP of empagliflozin 10 mg tablets or matching placebo are produced by the Glostrup Pharmacy as identical white capsules delivered to each patient as a container with 90 capsules. The Glostrup Pharmacy is a public pharmacy which is independent of the steering committee and study centres. The IP is produced and controlled in accordance with the requirements in the *Commission Directive 2003/94/EC of 8 October 2003* laying down the principles and guidelines of good manufacturing practice and in compliance with *Good Medical Practice* (*GMP*)*.* The allocation sequence is generated by Glostrup Pharmacy using computer-generated random numbers in blocks of 10. Treatment may be unblinded in medical emergencies during the study if the investigators deem it necessary. Unblinding during the study period may be made individually and is performed by telephone contact from the investigators to Glostrup Pharmacy, where the allocation sequence is stored. A copy of the allocation sequence is concealed in opaque, sealed envelopes which are stored in a locked cabinet in the sponsor’s office. Data analysis will be blinded to the investigators regarding primary, exploratory, and secondary endpoints (triple blinding).

### Study organization

#### Study centers and time schedule

Patients are recruited from specialized HF clinics at five sites in Denmark (Herlev-Gentofte Hospital, Odense University Hospital, Bispebjerg-Frederiksberg Hospital, Rigshospitalet, and Amager-Hvidovre Hospital). Screening, randomization, and protocol-specified assessments are performed at two sites (Herlev-Gentofte Hospital and Odense University Hospital). All assessments are performed at both sites, except RHC, which is only performed in patients randomized at Odense University Hospital and DXA-scan, and ^51^Cr-EDTA clearance and dobutamine stress-echocardiography, which are only performed in patients randomized at Herlev-Gentofte Hospital (Fig. 2). The study protocol and the used methods are routine procedures at the performing sites [40, 41]. At present (December 2018), 112 patients have been randomized and enrolment follows the planned schedule. It is expected that the last patient’s last visit will be in October 2019.

#### Steering committee

The steering committee consists of JJ, MO, CK, MKP, CT, IG, LK (chair), FG, EF, NEB, LV, JEM, and MS. The steering committee is responsible for the design, monitoring, reporting, and publication of the trial. Primary investigators are MS at Herlev-Gentofte Hospital and JEM at Odense University Hospital. The steering committee will have access to the final trial dataset.

#### Monitoring and data collection

Data will be collected and stored using electronic case report forms (eCRFs) constructed in the Research Electronic Data Capture (REDCap) system (Vanderbilt University ©2018). Corresponding source documents are stored at the experimental sites in accordance with the rules and regulations of the Danish Data Protection Agency to ensure confidentiality. The study is monitored by the GCP units at the University of Copenhagen and the University of Southern Denmark based on a specific monitoring plan. The GCP units are independent from the steering committee.

#### Ethics and adverse events

The safety of the randomized patients will be monitored continuously based on recording of AEs and severe adverse events (SAEs) from signing the informed consent form through four weeks after the end-of-study visit. The data will be collected and recorded on standardized forms at each contact. After the end-of-study visit, no planned contacts are performed but patients are instructed to contact the investigators if late-occurring AEs are suspected. These data are reported to the relevant authorities in accordance with applicable laws and International Conference of Harmonization Good Clinical Practice (ICH-GCP) guidelines. An independent endocrinologist is the unblinded data monitor and will evaluate the AEs and SAEs when half the patients are enrolled and can make the final decision to terminate the trial based on these safety data. Previously, no hypoglycemic events were observed when HF patients without T2D were included in a trial at Herlev-Gentofte Hospital evaluating the anti-glycemic drug Liraglutide [42]. Empagliflozin is approved for treatment of T2D. As the mechanisms behind the cardioprotective effects of empagliflozin are unknown, the steering committee finds it ethically acceptable to test active medication against placebo, instead of an active comparator. There are ongoing RCTs evaluating SGLT2 inhibitors in HFrEF patients both with and without T2D (Empagliflozin Outcome Trial in Patients with Chronic Heart Failure with Reduced Ejection Fraction [EMPEROR-Reduced], ClinicalTrials.gov Identifier NCT03057977; Study to Evaluate the Effect of Dapagliflozin on the Incidence of Worsening Heart Failure or Cardiovascular Death in Patients with Chronic Heart Failure [DAPA HF], ClinicalTrials.gov Identifier NCT03036124). The risk of significant side effects to empagliflozin is estimated to be modest. Compensation to those patients who suffer harm from study participation is set by the public Patient Compensation Association in Denmark.

#### Biobank

A research biobank is established in relation to the trial, where blood and urine samples are stored in coded form for later analysis of biomarkers. After the analyses declared in the protocol, the samples will be anonymized and the research biobank will be discontinued in accordance with the rules and regulations of the Danish Data Protection Agency. Patients are informed about the research biobank before signing the informed consent form.

#### Dissemination of results

The results of the study will be submitted to international peer-reviewed scientific journals, irrespective of their outcome, and the data will be made available to the public via EudraCT (www.clinicaltrialsregister.eu) and www.clinicaltrials.gov. Positive, inconclusive, and negative results will be presented. Furthermore, the results will be presented at scientific conferences as abstracts, oral presentations, and posters. The steering committee will assess authorship eligibility for the scientific papers related to the study based on the recommendations of the International Committee of Medical Journal Editors (ICMJE).

#### Conclusions and clinical implications

The anticipated results from ongoing randomized clinical trials will decide whether SGLT2 inhibitors will be a future treatment option in HFrEF patients. The Empire HF trial will complement these event-driven trials with mechanistic insight supporting clinicians and researchers in understanding the underlying mode of action of SGLT2 inhibitors including whether the observed effect on clinical outcomes is cardiac, renal, and/or metabolic, and whether SGLT2 inhibitors have an impact on patient-centered endpoints including physical activity and quality of life.

## Trial status

The study is currently recruiting and enrolling participants. Protocol version 5, 5 October 2018. Start of recruitment: 29 June 2017. Approximate date when recruitment will be completed: 31 October 2019.

## Additional file

Additional file 1: Checklist for the reporting of study protocols in accordance with the Standard Protocol Items: Recommendations for Interventional Trials (SPIRIT). (DOCX 50 kb)

## Data Availability

The datasets used and/or analyzed during the current study are available from the corresponding author on reasonable request.

## Abbreviations

(e)ECV: (Estimated) extracellular volume
(e)GFR: (Estimated) glomerular filtration rate
(e)PV: (Estimated) plasma volume
^51^Cr-EDTA: Chromium-51 labeled ethylenediamine tetraacetic acid
AE: Adverse event
ANCOVA: Analysis of covariance
CANVAS: Canagliflozin cardiovascular assessment study
CI: Cardiac index
CRT-D: Cardiac resynchronization therapy with defibrillator
CRT-P: Cardiac resynchronization therapy without defibrillator
DAPA HF: Study to evaluate the effect of dapagliflozin on the incidence of worsening heart failure or cardiovascular death in patients with chronic heart failure
DECLARE-TIMI 58: Dapagliflozin effect on cardiovascular events-thrombolysis in myocardial infarction 58
DXA: Dual-energy X-ray absorptiometry
eCRF: Electronic case report form
EMPA-REG OUTCOME: Empagliflozin, cardiovascular outcomes, and mortality in type 2 diabetes
EMPEROR-Reduced: Empagliflozin outcome trial in patients with chronic heart failure with reduced ejection fraction
EQ-5D-5 L: EuroQol 5-dimension 5-level
GCP: Good clinical practice
GMP: Good medical practice
HbA1c: Hemoglobin A1c
HF: Heart failure
HFpEF: Heart failure with preserved ejection fraction
HFrEF: Heart failure with reduced ejection fraction
hs-cTNI: High-sensitivity cardiac troponin I
ICD: Implantable cardioverter defibrillator
ICH-GCP: International conference of harmonization good clinical practice
ICMJE: International committee of medical journal editors
IP: Investigational product
IQR: Interquartile range
ITT: Intention-to-treat
KCCQ: Kansas City cardiomyopathy questionnaire
LV: Left ventricular
LVEF: Left ventricular ejection fraction
LV-GLS: Left ventricular global longitudinal strain
MR-proADM: Mid-region pro-peptide of adrenomedullin
NT-proBNP: N-terminal pro-brain natriuretic peptide
NYHA: New York heart association
OGTT: Oral glucose tolerance test
PCWP: Pulmonary capillary wedge pressure
REDCap: Research electronic data capture
RHC: Right heart catheterization
SAE: Severe adverse event
SD: Standard deviation
SGLT2: Sodium-glucose cotransporter 2
SPIRIT: Standard Protocol Items: Recommendations for Interventional Trials
T2D: Type 2 diabetes
UACR: Urine albumin to creatinine ratio
